# New Systemic Therapy Options for Advanced Sarcomas

**DOI:** 10.1007/s11864-012-0196-2

**Published:** 2012-06-04

**Authors:** Winette T. A. van der Graaf, Hans Gelderblom

**Affiliations:** 1 Department of Medical Oncology, Radboud University Nijmegen Medical Centre, PO Box 9101, 6500 HB Nijmegen, The Netherlands; 2 Department of Clinical Oncology, Leiden University Medical Center, PO Box 9600, 2300 RC Leiden, The Netherlands

**Keywords:** Sarcoma, GIST, Trabectedin, Pazopanib, Eribulin, Imatinib, Nilotinib, Masitinib, Regorafenib, Sorafenib, Denosumab, Sorafenib, Denosumab, Ridaforolimus, IGFR, cMET, ALK, Crizotinib

## Abstract

The outcome of patients with advanced soft tissue sarcomas (STS) has not improved much during the last decade. Apart from non-pleomorphic rhabdomyosarcoma, adjuvant chemotherapy has no standard role in high risk STS. In metastatic disease little progress has been made, but during recent years much effort has been put into the development of better clinical study protocols, with stratification of patients to at least the most common histological subtypes, preventing the dilution of potential treatment efficacy when measuring results over the total heterogeneous group of STS. The outcome of patients with advanced STS is however not only dependent on the introduction of new drugs, but also on the availability of dedicated sarcoma centers in which multidisciplinary teams with the input of all experts from different disciplines, such as pathology, radiology, nuclear medicine, surgery, orthopedics radiotherapy and medical oncology is present. Long delay, wrong histological diagnoses, under- and overtreatment are not in the favor of these patients, neither with regard to outcome, nor with respect to short- and long-term toxicity. Disappointedly, centralization is not a routine part of daily care of STS patients and their care givers. Patient advocacy groups are more and more aware of the relevance of treatment in centers of expertise and are active in guiding the patients to these hospitals. At the same time the sarcoma centers should be pro-active in putting patients into clinical trials, also for rare indications within the STS group, as only in this way a better outcome for this group of patients can be reached.

## Introduction

Soft tissue sarcomas (STS) are a rare and heterogeneous group of mesenchymal malignancies. They comprise 1–2 % of all maliganancies and encompass about 50 histological subtypes. The outcome of the disease is poor, with a median survival of about 1 year in case of metastatic disease. Given the rarity and the heterogeneity of the disease, improvement in outcome for STS as a whole is difficult to reach. However, with the better understanding of the biology of at least certain subtypes of STS and the opportunities of molecular diagnostics new therapeutic options are on the horizon and are currently being tested in clinical studies. Not surprisingly, most of the attention from pharmaceutical companies has always been put into the big four killers: lung-, colon-, breast- and prostate cancer, and the awareness of the importance of performing clinical studies in rare cancers was difficult to reach until 2000. At that time the breakthrough of the targeted agent imatinib in gastro-intestinal stromal tumors (GIST) showed the oncological world that even in rare diseases concepts of new treatments can be born [1]. Since then a tremendous amount of research has been put in understanding more of the biology, sensitivity and resistance of this type of cancer. Even the commonly used RECIST (Response Evaluation Criteria In Solid Tumours) became debatable in the medical treatment of GIST, and new criteria of response evaluation became necessary. Until now the CHOI criteria have been the most widely used in this respect [2, 3].

In metastatic STS first line doxorubicin is the most commonly used chemotherapeutic agent, either as a single agent or in combination therapy with ifosfamide. Whether combination therapy, apart from a higher chance of inducing a response, also leads to a better survival is still unknown. The results of the European Organisation of Treatment and Development of Cancer (EORTC) Soft Tissue and Bone Sarcoma Group (STBSG) 62012 study, expected in 2012, will hopefully give an answer to this important question. Ifosfamide is applied in many cases of STS, but the toxicity (especially bone marrow, nephrotoxicity and encephalopathy) of the most widely applied schedules is an important limiting factor, especially in the elderly. Depending on registration and reimbursement per country, also other chemotherapeutic agents can be given, mostly restricted to certain tumor types. Well-known examples are paclitaxel in angiosarcoma, as well as gemcitabine with or without docetaxel in other STS, and trabectedin in leiomyosarcoma and liposarcoma. In recent years some new, potentially promising drugs have been tested in STS and—to a limited extent—also in the rare group of bone sarcomas. Given the rarity of the disease, national and international collaborations in performing studies in dedicated sarcoma centers is essential to make any progress in STS, as well as in several types of bone sarcomas, such as Ewing sarcoma (ES), osteosarcoma, and other orthopedic tumors. Several national groups have been founded during recent years which all aim at exploring new drugs in various histological subtypes. Nevertheless, phase 3 studies are still scarce.

In 2002, Van Glabbeke et al. published results of the database of EORTC STBSG in which, for phase 2 studies in STS, potential active and non active drug results were distinguished, i.e. in pretreated patients active drugs had a progression-free rate (PFR) of 39 % and 14 % at 3 and 6 months, respectively, while inactive regimens had a PFR of 21 % and 8 %, respectively [4]. For first-line therapy, a 6-month PFR of more than 30–56 % (depending on histology) was defined as a reference value for drug activity; for second-line therapy, a 3-month PFR of more than 40 % would suggest a drug activity, and less than 20 % inactivity.

## Treatment

### New chemotherapeutic agents

#### Trabectedin

Trabectedin is an alkylating agent that binds to the DNA minor groove. It was originally isolated from the Caribbean sea squirt *Ecteinascidea turbinata*, but is now synthetically manufactured. In recent years trabectedin (ET-743, Yondelis) has been granted orphan drug status in many countries for locally advanced STS, refractory or unsuitable to receive standard chemotherapy, consisting of doxorubicin and ifosfamide. The standard dose and regimen is 1.5 mg/m^2^ over 24 hours every 3 weeks, given over a central line with dexamethasone premedication to decrease the severity of liver and bone marrow toxicity. Other toxicities include nausea and fatigue and in rare cases also rhabdomyolysis. Strict dose reduction schedules based on prior toxicity and in between cycle safety laboratory measurements of bone marrow function, renal and liver function and serum creatinine phosphokinase are mandatory and are to be adhered to by the treating physician’s team. Toxicity does not seem to be cumulative, even after more than ten cycles. The efficacy of trabectedin has been shown mainly for lipo- and leiomyosarcomas in numerous phase II and compassionate use programs, although other STS subtypes may also benefit equally from the drug, such as synovial sarcoma. Remarkable efficacy was observed in myxoid liposarcoma with an approximately 50 % response rate in a retrospective study [5] and a 24 % partial response rate and 13 % pathological complete remission rate in a recent prospective neoadjuvant phase II study [6]. Only one randomized study has so far been completed [7•]. This randomized phase II study comparing a weekly 3-hour with a 3-weekly 24-hour schedule favours the latter schedule with a median time to progression of 3.7 months. However the lack of a randomized phase III study has thus far been an omission in the clinical development path of this drug. Therefore we welcome the current ongoing first line EORTC/SARC phase IIB/III study (NCT01189253; “TRUST”) in patients with locally advanced or metastatic STS. This study investigates the feasibility of a 3-hour 3-weekly infusion versus the standard 24-hour infusion, and the approach with the best results is being further compared to 3-weekly doxorubicin in the phase III part of the study with PFS as primary endpoint.

#### Eribulin

Eribulin mesylate (E7389, Halaven) is a synthetic analogue of halichondrin B, which was originally isolated from marine sponges. Like taxanes it is a tubulin targeting agent. It has shown activity in breast and lung cancer. The activity of taxanes is restricted to certain subtypes of soft tissue sarcomas. Paclitaxel has shown activity in angiosarcoma, and docetaxel has only shown efficacy in combination with gemcitabine, predominantly in leiomyosarcoma. In a non-randomised multistrata and multicenter phase 2 study the efficacy of eribulin was evaluated [8]. Eribulin was administered in a dosage of 1.4 mg/m^2^ on days 1 and 8 of a 3-week cycle until progression or limiting toxicity. In line with other studies of EORTC, the progression-free rate at 12 weeks (PFR _12weeks)_ was taken as endpoint of the study. In the study of Schöffski et al. 128 patients participated and were stratified according to the histological subtype. The PFR _12weeks_ was 47 % for liposarcoma, 32 % for leiomyosarcoma, 21 % for synovial sarcoma and 19 % for other STS. Most serious toxicities (grades 3–4) were neutropenia, anemia, fatigue, febrile neutropenia, mucositis and sensory neuropathy, and 5 % had increased alanine aminotransferase serum levels. Based on this promising activity, currently, a phase 3 study is running in liposarcoma and leiomyosarcoma (NCT01327885).

### Targeted agents

#### Pazopanib

Pazopanib (GW786034; Votrient) is a synthetic indazolpyrimidine, which targets multiple kinases, including vascular endothelial growth factors (VEGFR1-3) and platelet derived growth factors (PDGFR). Recently, pazopanib had been registered for the treatment of advanced renal cell cancer. In a previous stratified phase 2 clinical trial in relapsed or metastatic soft tissue sarcoma (EORTC study 62043) the progression free rate at PFR _12weeks_ was 44 % in leiomyosarcoma patients, 49 % in synovial sarcoma patients, 39 % in patients with other types of STS, and 26 % in liposarcoma patients [9]. Based on this promising activity a phase 3 study was designed by EORTC STBSG, in which pazopanib was compared with placebo in a 2:1 fashion in patients with progressive non-adipocytic STS, who had had anthracyclines and standard treatment for STS in their country. This so-called PALETTE trial (EORTC study 62072) was conducted in EORTC centers together with centers worldwide, no crossover was allowed in the study [10••]. The median PFS was 4.6 months with pazopanib compared to 1.6 month with placebo (hazard ratio 0.31, 95 % CI 0.24–0.40; *P* < 0.0001). The final overall survival (OS) was 12.5 months with pazopanib versus 10.7 months with placebo (HR 0.86; 0.67–1.10; *P* = 0.25). Pazopanib was generally well tolerated. Most common adverse events were: fatigue, diarrhea, nausea, weight loss and hypertension. Although the driving factor of this multikinase inhibitor is unknown, pazopanib is seen as a welcome member in the family of active drugs in STS. Many studies with combination treatments are ongoing.

#### Regorafenib

Regorafenib (BAY73-4506) is an oral tyrosine kinase inhibitor targeting multiple receptors, including VEGFR1-2, PDGFR beta, FGFR1 and KIT, and multiple key players in the intracellular signal transduction pathways. The dosing schedule is 160 mg/day d1-21 every 4 weeks. The main toxicities include hand-foot skin reactions and hypertension. The drug was recently found to be active in a phase III study in third line metastatic colorectal cancer and has the longest progression free rate of any drug tested in a third line GIST study [11]. Recently, a phase III study in patients that failed prior treatment with imatinib and sunitinib was completed and results will be presented at the ASCO 2012 annual meeting.

#### Sorafenib

Sorafenib (Nexavar) is an oral tyrosine kinase inhibitor that, amongst others, blocks VEGFRs, KIT, PDGFRs and RAF. It was the first targeted agent found to be active against metastatic clear cell renal cancer and is also registered for non-resectable hepatocellular carcinoma. The standard dose is 400 mg twice daily and toxicities are comparable to other oral angiogenesis inhibitors. Sorafenib shows varying degree of activity in phase II studies in multiple sarcoma subtypes including third line GIST, angiosarcoma and osteosarcoma [12–14]. Despite encouraging progression free rates and some responses we have to realize that progression rate before entry into the studies was not clearly defined. This, together with the lack of phase III studies in the more frequent subtypes such as GIST, leaves us with the conclusion that sorafenib has not yet a clearly defined role in the treatment of sarcoma.

#### Nilotinib

Nilotinib (Tasigna) is an oral tyrosine kinase inhibitor that inhibits BCR-ABL, KIT and PDGFRs and is active in some imatinib resistant forms of KIT and less sensitive to some drug efflux pumps than imatinib. The drug was recently registered in CML and the therapeutic dose is 400 mg twice daily on a continuous schedule. The main toxicities include fatigue and gastrointestinal complaints. After promising activity in a phase I study [15] and a compassionate use study [16], both in imatinib and sunitinib resistant GIST, a phase III study was undertaken. In this study imatinib and sunitinib refractory or intolerant patients were randomized in a 2:1 ratio between nilotinib and best supportive care, that may include retreatment with imatinib or sunitinib [17•]. In the main analysis of this paper the median PFS was similar between the two arms. There was a discrepancy between investigator and central RECIST reading, emphasizing the drawbacks of RECIST-PFS as primary endpoint in these patients. In an unplanned post hoc analysis in true third line patients progressive on both prior imatinib and sunitinib, a more than 4 months survival benefit of nilotinib was observed, despite cross-over availability to nilotinib in the best supportive care arm. In retrospect, the design of this study has not been an optimal one, which, unfortunately, has negative consequences with regard to registration of this drug for this indication.

#### Masitinib

Masitinib (AB1010) is a novel oral tyrosine kinase inhibitor of c-KIT, PDGFR and Lyn, making it particularly interesting for inhibition of mast cells in systemic mastocytosis in dogs and humans. The drug has greater preclinical activity than imatinib in some KIT isoforms. The recommended dose is 7.5–12 mg/kg/d in a continuous schedule and main toxicities include rash, asthenia, diarrhea, nausea and muscle cramps, as with imatinib. In a recent first line phase II study in metastatic GIST the drug showed remarkable activity with a disease control rate of 97 % and an estimated PFS of 41.3 months [18]. Based on these results in first line treatment of GIST and based on promising activity in imatinib-resistant GIST patients in the phase I study [19], it was decided to start two randomised studies in metastatic GIST—a first line phase III study (NCT00812240) comparing masitinib to imatinib and a second line phase II study (NCT01506336) comparing mastinib to sunitinib.

#### Ridaforolimus

Ridaforolimus (AP23573, MK-8669, formerly deforolimus) is an inhibitor of mammalian target of rapamycin (mTOR), an integral component of the phosphatidyl 3-kinase (P13K)/AKT signaling pathway. Deregulation of the PI3K/AKT pathway is frequently observed in a variety of malignancies. In a multicenter, open-label, single-arm, phase II trial the antitumor activity of ridaforolimus in patients with distinct subtypes of advanced sarcomas was tested [20]. Patients with metastatic or unresectable soft tissue or bone sarcomas received ridaforolimus 12.5 mg administered as a 30-minute intravenous infusion once daily for 5 days every 2 weeks. The primary end point was clinical benefit response (CBR) rate (complete response or partial response or stable disease ≥ 16 weeks). A total of 212 patients were treated in four separate histological cohorts: primary bone sarcomas, leiomyosarcomas, liposarcomas, and other soft-tissue sarcomas. Sixty-one patients (28.8 %) of this pretreated population achieved CBR, with a median PFS of 15.3 weeks and a median OS of 40 weeks. RECIST confirmed response rate was 1.9 % (*n* = 4); three responses were observed in bone sarcomas. Adverse effects mainly consisted of fatigue, stomatitis, hypertriglyceridemia, anemia, rash, and nausea. A phase 3 study was designed in which patients, after initial chemotherapy in advanced sarcomas, were randomised between ridaferolimus and placebo as maintenance therapy. Results of this SUCCEED (Sarcoma Multi-Center Clinical Evaluation of the Efficacy of Ridaforolimus) study were reported at ASCO 2011 [21]. In this phase 3 study STS and bone sarcoma patients received ridaforolimus 40 mg orally for 5 days/week vs. placebo as maintenance therapy following stable disease or better response to prior chemotherapy. A total of 711 patients were randomised, and the median PFS increased from 17.7 weeks in the ridaforolimus arm compared with 14.6 weeks in the placebo arm (*P* < 0.001); no difference in overall survival was observed. Although an interesting concept of testing maintenance therapy in advanced sarcoma, the results of SUCCEED are not in favor of ridaforolimus given the minimal improvement in PFS and the non-negligible adverse events of this drug [22].

#### Denosumab

Denosumab (Xgeva) is a human antibody that inhibits RANK ligand (RANKL), a key mediator in osteclast-like giant cells and their precursors. Inhibition of RANKL by denosumab in giant cell tumours of bone was expected to inhibit bone destruction and giant cells. The drug is being administered subcutaneously and its toxicity is limited to sporadic acute phase reactions, hypocalcaemia and infrequently osteonecrosis of the jaw. The drug is being registered at a lower dose in bone metastases and in osteoporosis. The dose in studies in giant cell tumor of bone is 120 mg subcutaneously on day 1, 8, 15, 28 and then q 28 days. The activity of denosumab in giant cell tumour of bone is, as expected for this “Rolls Royce of giant cell dependent tumours,” astonishing. Virtually all patients experience pain reduction and predefined clinical efficacy of 86 % [23••]. Preliminary unpublished data suggest that surgery after denosumab treatment is easier than without due to the formation of a calcified boundary around the tumor. Currently the activity of the drug is being investigated in a larger phase II study (NCT00680992) in two cohorts: non-resectable and metastatic disease. This drug is certainly one of the most active drugs in oncology in the last decades and deserves registration for this indication in our opinion.

### Imatinib: “old” drug, new indications

#### Imatinib adjuvant

Imatinib (Glivec/Gleevec) was the first tyrosine kinase inhibitor that was registered in oncology, initially for CML and then for metastatic GIST. The standard dose is 400 mg once daily for metastatic GIST, and twice daily for exon 9 mutated GIST. Recently the landscape of adjuvant treatment for GIST has changed. After the first adjuvant 1-year imatinib study in GISTs > 2 cm that showed a PFS benefit but no OS advantage [24], a second adjuvant study in high risk GIST changed the daily practice. The SSG study showed not only a PFS but also an OS advantage in c-KIT positive high risk GIST patients treated with imatinib for 3 years compared to 1 year [25••]. International guidelines were adapted to incorporate 3 years of adjuvant imatinib as the gold standard, except for known imatinib resistant mutations, such as PDGFR D842V mutated GIST.

#### Imatinib-PVNS

The role of imatinib in diffuse type tenosynovial giant cell tumour (TGCT), also known as pigmented villonodular synovitis (PVNS), was first described in a case report by Blay et al. [26]. The proposed mechanism of action is as follows: this benign, but sometimes recurrent and then invalidating tumour, overexpresses colony stimulating factor 1 (CSF1) that may result from a t(1 ;2) translocation. Active CSF1 attracts inflammatory cells that in its turn express CSF1R (receptor) through a paracrine or “landscape” effect. Imatinib inhibits CSF1R (albeit in the high nanomolar range) and therefore is an interesting drug for this disease. In a retrospective study of 29 patients imatinib displayed interesting activity (overall response rate 19 % and 74 % stable disease) [27]. However the benefits of alleviating morbidity must be balanced against potential toxicity of chronic imatinib therapy in this relatively young patient population. In this study the median age was 41 years. Currently a prospective phase II study is underway studying the effect of nilotinib in this patient population (NCT01261429). More specific CSF1R inhibitors may be needed for this disease.

#### Imatinib-Chordoma

Chordomas that are not amenable to radiotherapy or surgery can lead to severe morbidity, and therefore systemic treatment options are being explored. Recently, promising results of a phase II study were published [28]. Based on the fact that chordomas express the PDGFRs and based on some case reports, imatinib was the candidate to test in this study. Patients with progressive chordomas received 800 mg/d until progression. The response rate was 2 % with a 64 % clinical benefit rate as defined by absence of progression at 6 months. Due to the rarity of this disease probably no randomised studies will be undertaken to fully understand the effect of imatinib. These results indicate that in the absence of other treatment options or clinical studies imatinib is a reasonable treatment option in symptomatic progressive patients.

#### Imatinib-Desmoid

Desmoid type aggressive fibromatoses are rare benign, but locally aggressive tumours, sometimes associated with familial adenomatous polyposis. When desmoid are unresectable systemic treatment is often initiated with anti-estrogens, NSAIDs or even chemotherapy. The effect of these treatments are difficult to judge for several reasons. First, rare spontaneous regressions may overestimate treatment effect; second, again no clearly defined growth rate before start of treatment is available in this often indolent disease; and third, prospective studies are scarce and randomised studies are lacking. Against this background the recently published phase II study of imatinib in this disease, showing an objective response rate of 6 % and 1-year progression free survival of 66 % is promising, but should be interpreted with care [29].

#### Imatinib-DFSP

Dermatofibrosarcoma protuberans (DFSP) is a dermal sarcoma that is frequently characterized by an activated platelet derived growth factor receptor beta (PDGFR-B) as a result of a translocation between chromosome 17 and 22. This tumor rarely metastasizes, mainly in case of fibrosarcomatous (FS) progression, and has a tendency to recur after surgery of large lesions or after limited surgery at difficult locations such as the head and neck area. As imatinib inhibits PDGFR-B signal transduction multiple studies were undertaken to study the effect of this drug in this rare disease entity. The combined results of two uncompleted phase II studies of EORTC and SWOG show a clear benefit of imatinib with a high response rate approaching 50 %, including DFSP with FS progression, regardless of the dose (400 mg or 800 mg daily) [30].

### Emerging treatments

#### IGF

The insulin like growth factor-1 receptor (IGF-1R) pathway transduces extracellular signals intracellularly to mediate cell proliferation, growth, and survival. IGF-1R is activated on engagement by the growth factor ligands IGF-1 and IGF-2, resulting in receptor autophosphorylation. IGF-1R activity is also regulated by six IGF binding proteins, which lead to either promote or antagonize IGF-1R signaling by binding with IGF ligands in the circulation. This induces activation of multiple signaling cascades, including the PI3K/AKT/mTOR pathway which, when aberrantly activated, promotes the oncogenic phenotype. Several lines of evidence have suggested that IGF-1R signaling is critical to the biology of, for example, Ewing sarcoma (ES) [31, 32]. Olmos reported about promising effects of anti-IGF1R antibody figitimumab (CP751871) in a phase 1 study with refractory sarcomas [33]. Since then a couple of studies were executed with IGF-1R antibodies. Pappo et al. published about 115 patients who received the monoclonal IGF-1R antibody R1507 either at doses of 9 mg/kg intravenously once a week or 27 mg/kg intravenously every three weeks [34]. The overall complete and partial response was 10 %, with a median duration of response of 29 weeks (range, 12–94 weeks). The median OS was 7.6 months (95 % CI, 6–9.7 months). Ten of 11 responses were observed in patients who presented with bone tumors (*P* = 0.016). At the same time a phase 1/2 study was reported with figitimumab in patients with ES, and other bone and STS sarcomas [35]. In the phase 2 part of the study, 107 patients with ES received figitumumab at 30 mg/kg every 4 weeks for a median of 2 cycles (range, 1–16). Of 106 evaluable and in general heavily pretreated patients, 15 had a PR (overall response rate, ORR 14.2 %) and 25 had stable disease. Median OS was 8.9 months. Importantly, pretreatment free IGF-1 levels discriminated between short and long OS. Simultaneously, Fleuren et al. showed in ES and osteosarcoma xenografts visualization with (111)In-R1507 immuno-SPECT of membranous IGF-1R expression and target accessibility [36]. Although in this way non-invasive prediction of therapy response to IGF-1R therapy (e.g. R1507) could be developed also for clinical use, unfortunately quite a couple of pharmaceutical companies have decided to stop the development of IGF1-R antibody programs. Results of IGF-1R combination studies [37] are promising and studies with (dual) tyrosine kinase inhibitors are urgently awaited.

#### ALK

Inflammatory myofibroblastic tumor (IMT) is a distinctive mesenchymal neoplasm characterized by a spindle-cell proliferation with an inflammatory infiltrate. Approximately half of IMTs carry rearrangements of the anaplastic lymphoma kinase (ALK) locus on chromosome 2p23, causing aberrant ALK expression. Recently, a lasting partial response to the ALK inhibitor crizotinib (PF-02341066), known for its striking clinical activity in non-small-cell lung cancers with *EML4-ALK* rearrangements, was reported in a patient with ALK-translocated IMT, as compared with no observed activity in another patient without the ALK translocation [38•]. These results support the dependence of ALK-rearranged tumors on ALK-mediated signaling and warrants further study in IMTs. Recently, in 189 paraffin-embedded rhabdomyosarcoma (RMS) tumor specimens from 145 patients ALK aberrations on genomic and protein levels were found to occur frequently and were related to disease progression and outcome [39]. Therefore, the oncogenic role of ALK and the potential targeted treatment with ALK inhibitors in RMS deserve further research.

#### MET

Hepatocyte growth factor/scatter factor (HGF/SF) and its receptor, c-Met, have been implicated in the growth and progression of a variety of solid human tumors. Inhibition of HGF/SF:c-Met signaling may provide a novel therapeutic approach for treating human tumors. Recently it was shown that in clear cell sarcoma (CCS), known for its chemoresistance, blocking c-Met activity with a small-molecule inhibitor (SU11274) or a neutralizing antibody to its ligand HGF/SF (AMG 102) CCS cell growth was significantly reduced in culture [40]. Also in leiomyosarcoma interrupting autocrine and/or paracrine HGF/SF:c-Met signaling with AMG 102 had profound antitumor effects [41].

Alveolar soft part sarcoma (ASPS) is a rare chemoresistant STS which is characterized by the presence of a specific chromosomal translocation encoding the chimeric transcription factor (ASPL-TFE3) that activates expression of MET. In eight patients immunohistochemical staining demonstrated 100 % TFE3 positivity and 75 % MET positivity with a strong association between TFE3 expression and MET positivity with correlation coefficient of 0.808 (*P* = 0.02) [42]. The high expression of MET in ASPL-TFE3 (+) ASPS may underscore the potential role of targeted agents against MET in ASPS.

In 2012, the EORTC study 90101 “CREATE” (NCT01524926) will start. This cross-tumoral phase 2 study will address the issue of ALK and Met driven tumors with crizotinib (PF-02341066). Four strata for sarcomas will be part of CREATE: IMT, alveolar RMS, CSS and ASPS.
